# Endoplasmic reticulum as a target in cardiovascular diseases: Is there a role for flavonoids?

**DOI:** 10.3389/fphar.2022.1027633

**Published:** 2023-01-10

**Authors:** Kimia Keylani, Fariba Arbab Mojeni, Amirmohammad Khalaji, Asma Rasouli, Dlnya Aminzade, Mohammad Amin Karimi, Pantea Majma Sanaye, Nazanin Khajevand, Nasrin Nemayandeh, Mohadeseh Poudineh, Mehdi Azizabadi Farahani, Mohammad Ali Esfandiari, Sepehr Haghshoar, Ali Kheirandish, Erfan Amouei, Amir Abdi, Arash Azizinezhad, Afshin Khani, Niloofar Deravi

**Affiliations:** ^1^ School of Pharmacy, Shahid Beheshti University of Medical Sciences, Tehran, Iran; ^2^ Student Research Committee, School of Medicine, Mazandaran University of Medical Sciences, Sari, Iran; ^3^ School of Medicine, Tehran University of Medical Sciences, Tehran, Iran; ^4^ School of Medicine, Zanjan University of Medical Sciences, Zanjan, Iran; ^5^ Student Research Committee, School of Medicine, Shahid Beheshti University of Medical Sciences, Tehran, Iran; ^6^ School of Medicine, Shahid Beheshti University of Medical Sciences, Tehran, Iran; ^7^ School of Pharmacy, Zanjan University of Medical Sciences, Zanjan, Iran; ^8^ Drug and Food Control Department, Faculty of Pharmacy, Tehran University of Medical Sciences, Tehran, Iran; ^9^ Amol Faculty of Paramedical Sciences, Mazandaran University of Medical Sciences, Sari, Iran; ^10^ Student Research Committee, Faculty of Medicine, Guilan University of Medical Sciences, Rasht, Iran; ^11^ Faculty of Pharmacy, Cyprus International University, Nicosia, Cyprus; ^12^ Student Research Committee, Faculty of Pharmacy, Mazandaran University of Medical Sciences, Sari, Iran; ^13^ Research Center for Prevention of Cardiovascular Disease, Institute of Endocrinology and Metabolism, Iran University of Medical Science, Tehran, Iran; ^14^ Student Research Committee, School of Medicine, Tehran Medical Sciences, Islamic Azad University, Tehran, Iran; ^15^ Universal Scientific Education and Research Network (USERN), Tehran, Iran; ^16^ Department of Cardiovascular Disease, Cardiovascular Research Center, Mazandaran University of Medical Sciences, Sari, Iran

**Keywords:** endoplasmic reticulum stress, flavonoids, quercetin, kaempferol, cardiovascular diseases

## Abstract

Flavonoids are found in natural health products and plant-based foods. The flavonoid molecules contain a 15-carbon skeleton with the particular structural construction of subclasses. The most flavonoid’s critical subclasses with improved health properties are the catechins or flavonols (e.g., epigallocatechin 3-gallate from green tea), the flavones (e.g., apigenin from celery), the flavanones (e.g., naringenin from citrus), the flavanols (e.g., quercetin glycosides from berries, onion, and apples), the isoflavones (e.g., genistein from soya beans) and the anthocyanins (e.g., cyanidin-3-O-glucoside from berries). Scientific data conclusively demonstrates that frequent intake of efficient amounts of dietary flavonoids decreases chronic inflammation and the chance of oxidative stress expressing the pathogenesis of human diseases like cardiovascular diseases (CVDs). The endoplasmic reticulum (ER) is a critical organelle that plays a role in protein folding, post-transcriptional conversion, and transportation, which plays a critical part in maintaining cell homeostasis. Various stimuli can lead to the creation of unfolded or misfolded proteins in the endoplasmic reticulum and then arise in endoplasmic reticulum stress. Constant endoplasmic reticulum stress triggers unfolded protein response (UPR), which ultimately causes apoptosis. Research has shown that endoplasmic reticulum stress plays a critical part in the pathogenesis of several cardiovascular diseases, including diabetic cardiomyopathy, ischemic heart disease, heart failure, aortic aneurysm, and hypertension. Endoplasmic reticulum stress could be one of the crucial points in treating multiple cardiovascular diseases. In this review, we summarized findings on flavonoids’ effects on the endoplasmic reticulum and their role in the prevention and treatment of cardiovascular diseases.

## 1 Introduction

Flavonoids are present in all photosynthesizing cells, thus occurring commonly in the plant and they describe as the largest class of polyphenols. They have been discovered in flowers, stems, nuts, vegetables, seeds, and fruit in addition to honey, tea, propolis, and wine, representing an ordinary human food component (Cushnie and Lamb, 2005). These combinations are comprised of 15 carbon atoms in their chemical structure. The main structural part of flavonoid combinations is the flavone nucleus consisting of two benzene rings conjoined *via* a heterocyclic pyrene ring (Serafini et al., 2010). Flavonoids are divided into flavans, anthocyanidins, flavonols, flavanones, isoflavones, and flavones (Ponte et al., 2021). Flavonoids are being used as epigenetic modifiers and anti-cancer agents in breast cancer (Mahmoud et al., 2019). Moreover, they are common products of plant derivatives that are reportedly documented to be therapeutically effective phytochemicals against considerable illnesses including oxidative stress, inflammatory disorders (cardiovascular diseases, neurodegenerative disorder), and malignancies. One of the diseases that has been proven to affect flavonoids is cardiovascular disease. For instance, cardiomyopathy, vasoconstriction, hypertension, heart failure, arrhythmia, thrombosis, inflammation, and Atherosclerosis are cardiovascular diseases affecting flavonoids (Mahmoud et al., 2019). The endoplasmic reticulum (ER) plays an important role in the metabolism of lipids, transmembrane proteins, maturation, folding, and synthesis of transmembrane and secretory proteins in most eukaryotic cells (Giordano et al., 2014). ER disorders, particularly ER stress, is a pathophysiological reaction concerned with lipid metabolism and cardiovascular injury. Thus, suppression of ER stress may refine lipid metabolic disease and decrease cardiovascular risk (Li et al., 2018a). Human and animal samples of atherosclerosis lesions have provided specific proof that ER stress exists in atherosclerotic plaques, especially at the advanced level (Liu et al., 2016). This narrative review may open avenues for investigation of the flavonoids’ properties in the decrease of ER stress in treating cardiovascular diseases.

## 2 Methods

In this study, we searched various online databases including Google Scholar, PubMed, Web of Science, and EMBASE to find all articles related to ER as a target in cardiovascular diseases and flavonoids’ role in ER stress, published from January 2003 until March 2020. No limitation has been applied to the language of the articles. The search strategies used for each database were independently designed and MeSH terms were also included. The keywords were:

#1 Flavonols #2 Quercetin #3 Kaempferol #4 Fisetin #5 Flavanones #6 Naringenin #7 Isoflavones #8 Genistein #9 Flavones #10 Apigenin #11 Baicalein #12 Luteolin #13 Wogonin #14 Nobiletin #15 Total flavonoids #16 Thrombosis #17 Myocardial infarction #18 Myocardial ischemia/reperfusion #19 Autoimmune myocarditis #20 Cardiomyocyte apoptosis #21 Cardiac inflammation #22 Arrhythmia #23 Diabetic cardiomyopathy #24 Hypertrophic cardiomyopathy #25 Atherosclerosis #26 Hypertensive heart disease #27 Cardioprotective #28 Endoplasmic reticulum stress.

We selected relevant studies by screening the title and abstracts of the articles. Afterward, the reference lists of included articles were also surveyed to identify any further studies.

## 3 Role of ER in cardiovascular diseases

Increasing physiological and pathological damage, including changes in intracellular calcium, genetic or environmental damage, oxidative stress, and glycosylation, could disturb ER’s steady state leading to the ER lumen not folding and misfolded proteins. This condition is known as ER stress (Paschen and Frandsen, 2001; Schröder and Kaufman, 2005; Zhang et al., 2015). ER stress is associated with autophagy and the immune response to pathogens. Targeting ER stress and the unfolded protein response (UPR) with small molecules is considered a promising therapy for treating various diseases such as neurodegeneration, cancer, metabolic diseases, stroke, and heart diseases (Valenzuela et al., 2018). Interestingly, in some diseases (e.g., pancreatic *β* cells in diabetes mellitus), it may be beneficial to suppress ER stress and/or block UPR, whereas in other diseases (e.g., viral infections), inducing ER stress and/or increase the UPR may be necessary for a therapeutic effect (Lin et al., 2008).

Cardiomyocyte hypoxia in addition to chronic and acute inflammatory reactions, which are present in heart failure, might cause ER stress (Dickhout et al., 2011). ER stress and the UPR will result in cardiac hypertrophy and heart failure by increasing PERK and EIF2A–ATF4–CCAAT–CHOP. Cardiovascular stress prompts ER stress and activates the UPR response to restore ER homeostasis. As an adjustable mechanism, the PERK–PI3K–AKT cascade inhibits apoptosis. The PERK activates autophagy and interrupts the generation of reactive oxygen species (ROS) which can prevent ROS-mediated cardiomyocyte injury; it also induces autophagy by activating eukaryotic initiation factor 2A (EIF2A) and prompts the activation of activating transcription factor 4 (ATF4), which leads to initiation of various types of autophagy (Ren et al., 2021). ATF4 triggers C/EBP homologous protein (CHOP) to induce various biological responses such as apoptosis and inflammation. Inositol-requiring protein 1*α* (IRE1α) also activates several cell signaling systems which lead to the inhibition of apoptosis and reduction of ROS levels. If the adaptive UPR fails to stabilize ER homeostasis, it results in cardiomyocyte apoptosis (Ren et al., 2021). The ER is an organelle providing multiple functions, including lipid biosynthesis, calcium storage, and protein folding and processing. Due to its various functions, ER stress has been highlighted as an effective regulator of cardiovascular diseases (Hong et al., 2017). Herein, we review ER stress in known cardiovascular disease.

### 3.1 ER stress in ischemic heart diseases

Zhang et al. (2017a) demonstrated that the key ERS molecules (ATF4 and CHOP) are stimulated in ischemic heart disease, and CHOP can induce the expression of proapoptotic proteins during ERS, accelerating the process of disease. A study by Xin et al. (2011a) showed that the PERK pathway was also activated due to the expression of phosphorylated eIF2α, and also ATF4 proteins were increased. Additionally, GRP78, the central regulator of ER function and UPR network modulator was also induced in the ischemic myocardium. Another study by Belmont et al. (2010) showed that Derl3 which encodes a protein involved in ERAD (Endoplasmic-reticulum-associated protein degradation), was induced by ATF6. Derl3 was also induced in the infarct border zone in a mouse model of myocardial infarction, and by simulated ischemia in cultured cardiac myocytes. It was found that overexpressing Derl3 reduces long-term ER stress response signaling and cell death.

### 3.2 ER stress in heart failure

Heart failure is a progressive disorder of the myocardial remodeling (Chin et al., 2011). Sigma-1 receptor (Sig-1R) is a chaperone receptor located at ER that acts as a dynamic pluripotent modulator regulating cellular pathophysiological processes. Sig-1R inhibition promotes autophagy in cardiomyocytes under oxidative stress conditions causing heart failure (Ortíz-Rentería et al., 2018). GRP78 protein and ER stress-related apoptotic pathways, CHOP, caspase-12, and JNK1 significantly increased in animal models of heart failure (Xin et al., 2011b). It has been suggested that TRB3 acts as an important role in cell death in relation to CHOP where CHOP is also induced by other stress signals; Under prolonged ER stress, induced TRB could lead to cell death and heart failure (Ohoka et al., 2005). SERCA3f is also upregulated in heart failure, and overexpression of the Ca^2+^ pump, increases the XBP1 splicing and Grp78 expression; This demonstrated that SERCA3f is probably a UPR effector and participates in the ER stress in human heart failure (Safiedeen et al., 2017).

### 3.3 ER stress in myocardial hypertrophy

Nogo-B is a conserved ER protein that is a player in maintaining the ER structure (Park et al., 2017; Weng et al., 2018). By activating the PERK/ATF4 signaling pathway, Nogo-β is prevented, resulting in myocardial hypertrophy (Li et al., 2018b). Evidence reveals that the expression of Nogo-β and ERS markers (such as p-PERK, ATF4, p-IRE1, and XBP1s) are significantly upregulated in transverse aortic constriction (TAC)-induced hypertrophic myocardium. In addition, MFN2 is known for its role in mitochondrial fusion. It is localized at ER and mitochondrial membranes and forms a hetero- or homo-dimer with mitofusin-1 (MFN1) or another MFN2 in the outer mitochondrial membrane (Choi et al., 2006; Eura et al., 2006). Downregulation of MFN2 has been found in rat models of cardiac hypertrophy (Sun et al., 2019).

### 3.4 ER stress in arrhythmias

Under prolonged ER stress, the ER could increase Ca^2+^ release, which activates cell apoptosis and also induce triggered activity that can cause cardiac arrhythmias (Safiedeen et al., 2017). A study by Liu et al. (2014) showed that ER stress is involved in the occurrence of ventricular arrhythmias in primary cardiomyocytes. ER stress markers including GRP78 and p-PERK were significantly elevated in the diabetic cardiomyopathy. Moreover, GRP78 expression and PERK phosphorylation elevations were found in diabetic myocardial infarcted hearts, indicating that the hyper-activation of ER stress-mediated PERK signaling was along with post-myocardial infarction ventricular arrhythmias in diabetes ones. It was reported that ER stress was hyperactivated through the RAGE (receptor for advanced glycation end products)/ROS pathway in myocardial infarcted hearts exposed to advanced glycation end products (Liu et al., 2021).

### 3.5 ER stress in atherosclerosis

Atherosclerosis is a chronic inflammatory disease in which inflammatory signaling pathways are involved (Ross, 1999; Libby et al., 2002; Tuttolomondo et al., 2012). Three ER stress sensors, PERK, IRE1, and ATF6, could all induce specific inflammatory responses through the UPR, especially in macrophages and ECs (Yang et al., 2020). PERK pathway results in phosphorylation of the inhibitor of nuclear factor-*κ*B (NF-*κ*B), I*κ*B, in which I*κ*B kinase is involved. This leads to the activation of genes involved in downstream pathways of inflammation, such as those that encode the cytokines TNF-*α* and interleukin (IL-1). The ATF6 pathway also triggers the NF-*κ*B pathway (Deng et al., 2004; Yamazaki et al., 2009; Hotamisligil, 2010). In addition, the IRE1 pathway induces the elevation of thioredoxin-interacting protein (TXNIP), which activates the NLRP3 inflammasome (Lerner et al., 2012). The activated form of NLRP3 promotes the secretion of IL-1*β* and IL-18 and leads to an inflammatory response (Martinon et al., 2002; Martinon and Tschopp, 2007; Takahashi, 2014). CHOP is also upregulated by the UPR in the ER, along with the progression of atherosclerosis in the aorta (Joo et al., 2015). Moreover, ER stress significantly stimulates macrophage lipid accumulation (Huang et al., 2018). The PERK signal activates GSK-3α expression. A study by Martin et al. (2005) showed that inhibition of GSK-3α remarkably reduces ER stress-induced free cholesterol levels and inhibits the expression of genes that regulate lipid and cholesterol metabolism, including fatty acid synthase, sterol element-binding protein-1 C (SREBP-1C), etc. Therefore, ER stress and PERK signal could trigger lipid metabolism which leads to atherosclerosis.

## 4 Current drug targets of ER for prevention or amelioration of cardiovascular diseases

ER stress significantly facilitates the development and occurrence of cardiovascular diseases. Various medications, including antihypertensive drugs, anti-glycemic drugs, lipid-lowering drugs, hormones, chemicals, herbal medicines, and other drugs, are used to target ER stress, which leads to preventing or alleviating cardiovascular diseases. Previous research has shown that Angiotensin II can cause ER stress (Wang et al., 2015). When ER stress arises, the UPR, a complex cellular signaling network, may be triggered, activating reparative signaling that includes PERK, eIF2, ATF3, and ATF6. For instance, Enalapril controls hypertension by blocking the PERK/eIF2α/ATF3/ATF6 pathway (Zhou et al., 2015a).

It is known that ER stress contributes to cardiotoxicity and heart conditions that could eventually lead to heart failure. Several molecular markers of ER stress, including GRP78, PERK, eIF2a, and CHOP, have been identified in the research to be expressed. Comparing the phosphorylated levels of eIF2a and PERK in patients with heart failure to controls, a considerable activation of the PERK to the eIF2a arm of the stress response was discovered. Metoprolol and propranolol as b-AR blockers suppress ER stress in cardiac hypertrophy and heart failure by blocking the GRP78/CHOP/XBP-1 pathway (George et al., 2011; Ni et al., 2011).

ER stress has a significant role in the initiation and progression of atherosclerosis (Tabas, 2010). ER stress indicators were found in atherosclerosis-prone portions of arterial regions susceptible or resistant to atherosclerosis separated from non-atherosclerotic pigs (Civelek et al., 2011). Atorvastatin affects atherosclerosis by inhibiting BIP/PERK/eIF2α/CHOP pathway (Song et al., 2011; Jia et al., 2012; Ivanova and Orekhov, 2016). Additionally, studies suggest that by inhibiting the PERK/eIF2/caspase-3 pathway, Atorvastatin may prevent apoptosis and, reduce cell damage (Yang and Hu, 2015).

Reports indicate that excessive glucose, improper fat acid metabolism, lipid buildup, and ROS increase cause diabetic cardiomyopathy by ER stress (Frustaci et al., 2016). Adding Metformin can prevent systolic and diastolic blood pressure by inhibiting ER stress markers including p-eIF2, GRP78, and XBP1s. Through the upregulation of p-eIF2, protein disulfide isomerase, and XBP1 mRNA expression, AMPK2 deletion causes ER stress progression. However, metformin lowers blood pressure *via* activating AMPK and inhibiting angiotensin-induced ER stress. Therefore, by boosting the phosphorylation of phosphoprotein, which was thought to be a key mechanism in the treatment of hypertension, metformin-activated AMPK2 may reduce ER stress (Hundal et al., 2000; Duan et al., 2017). Another anti-glycemic medication with cardioprotective effects is Empagliflozin. The Effects of Empagliflozin on diabetes and cardiomyopathy are *via* preventing the GRP78/CHOP/ATF4/caspase-12 signaling pathway (Zhou and Wu, 2017). Studies have demonstrated that empagliflozin enhanced cardiac functioning, decreased cardiomyocyte apoptosis, and decreased the expression of ER stress indicators such as Grp78, CHOP, and cleaved caspase-12 in proteins from the cardiomyocyte (Campeau and Leask, 2022).

One of the anti-anginal medications that are used commonly, is nicorandil. In addition to its vasodilator activity, it has been shown that it boosts fibrinolytic ability, protects the myocardium against thrombus formation, and promotes angiogenesis. Recent research has linked the development of atherosclerosis to ER stress (Thorp et al., 2009). A long-term nicorandil treatment significantly reduced the increased expression of the ER stress markers in atherosclerotic lesions. These findings imply that nicorandil’s beneficial effects are mediated by decreased ER stress. Following nicorandil treatment, the extent of the atherosclerotic lesion and plaque necrosis were significantly reduced. In atherosclerotic lesions, nicorandil significantly decreased the expression of the endoplasmic reticulum stress markers CHOP and GRP78. In cultured THP-1 macrophages, nicorandil significantly reduced tunicamycin-induced CHOP upregulation (Izumiya et al., 2011).

The vitamin D receptor (VDR), primarily expressed in the cardiovascular system, can control cardiovascular function. According to studies, VDR activation prevents ER stress-induced apoptosis, which protects against myocardial damage. Additionally, it has been shown that myocardial ischemia/reperfusion injury upregulates the expression of VDR. Curiously, calcitriol and paricalcitol, among other natural and synthetic agonists, activate VDR, significantly reducing infarct size and enhancing cardiac function. By decreasing the expression of CHOP and activating caspase-12, it has been shown that activating VDR limits the expression of ERS-relative protein, thereby alleviating myocardial ischemia/reperfusion injury (Pu et al., 2013; Yao et al., 2015; Haas et al., 2016). Through the inhibition of ER stress, Melatonin inhibits the evolution of cardiomyopathy. The pineal gland is the primary organ that secretes the endogenous chemical melatonin. Inhibiting ER stress-induced apoptosis is how Melatonin protects the myocardium. According to studies, Melatonin can reduce the expression of ER stress indicators in cardiac tissue, including CHOP, GRP78, PERK, and ATF6. It can also lower the expression of apoptosis-related markers like Bcl-2 and reverse the production of caspase-3 and Bcl-2 (Xiong et al., 2018).

Besides the drugs mentioned above, some herbal medicines that target ERS, have cardioprotective effects. Hypertension is linked to hyperhomocysteinemia or high homocysteine levels. Homocysteine induces ER stress in endothelial cells (Sutton-Tyrrell et al., 1997). Thus, ER stress plays a significant role in vascular dysfunction during hypertension. Consumption of Black tea is associated with reduced expressions of ER stress markers such as phosphorylated elF2α at ATF3 and cleaved ATF6, induced by hyperhomocysteinemia (San Cheang et al., 2015). Salidroside is another herbal remedy that protects endothelium cells from homocysteine-induced damage. Salidroside, an active ingredient in Rhodiola Rosea, has been shown to possess antioxidant properties. The underlying mechanisms of inhibition of Hcy-induced endothelial cells regulate the ER-stress pathway by down-regulating phosphorylation of PERK or IRE1, as well as the binding protein (Bip) and CHOP (Zhu et al., 2017a). Paeonia suffruticosa has been widely utilized in traditional oriental medicine for thousands of years and has a number of medicinal uses. Paeonia Suffruticosa’s root bark’s primary component is Paeonol (Zhang et al., 2019a). In a study, the vascular protective effects of chronic treatment with Paeonol have been explored. They found that using Paeonol preserved endothelial function and normalized blood pressure by inhibiting ER stress markers including GRP78, ATF6, and p-eIF2α (Choy et al., 2017; Chen et al., 2018).

Another chemical compounds that can affect ER stress are Flavonoids (Arumugam et al., 2012; Zhang et al., 2013a; Qin et al., 2016; Mahmoud et al., 2019). Flavonoids are used widely for this purpose, and this review aims to summarize the effects of this pharmaceutical category as a therapeutic agent in cardiovascular diseases. Table 1 summarizes the data of the studies on the effect of flavonoids on ERS inhibition in treating cardiovascular diseases.

**TABLE 1:** Mechanisms of flavonoids included in cardiovascular diseases.

Author (year)	Study design	Flavonoid	Dose	Duration	Cardiovascular disease	Outcomes	Chemical structure
Cai et al. (2015)	*In vitro*	Quercetin	0–20 μM	24 h	Atherosclerosis	• Prevents apoptosis	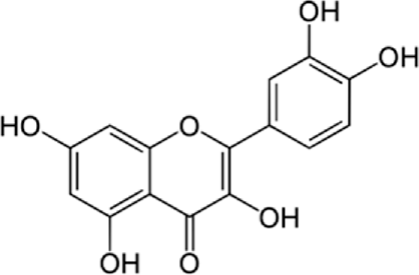
						• Prevents lipid accumulation in RAW264.7 macrophages	
Chang et al. (2021)	*In vitro*	Quercetin	50, 100, 150, 200, and 250 mg/L	Every 2 days	Cardiomyocyte apoptosis	• Regulates mitophagy	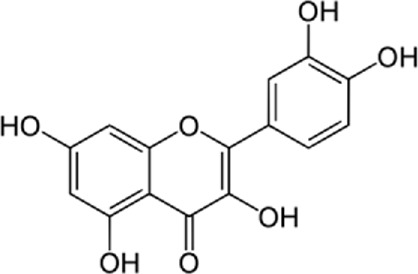
						• Regulates ER stress	
Zhang et al. (2017b)	*In vivo*	Nobiletin	50 mg/kg	4 weeks	Cardiac hypertrophy	• Inhibits oxidative stress	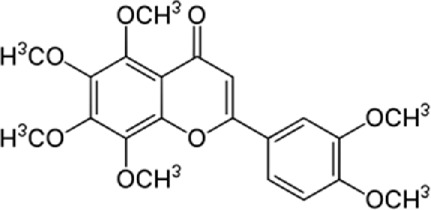
						• Inhibits ER stress	
						• Regulates ER stress	
Arumugam et al. (2012)	*In vivo*	Quercetin	10 mg/kg/day	28 days	Experimental autoimmune myocarditis	• Suppression of both the mitogen-active protein kinases (MAPK) and the myocardial endothelin-1	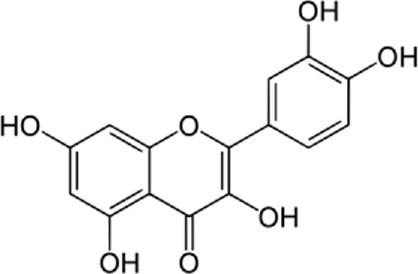
						• Suppression of oxidative and ER stress	
						• Cardioprotection against experimental autoimmune myocarditis	
Jasuja et al. (2012)	*In vivo*	Quercetin-3-O-rutinoside	0–50 mg/kg	90 min	Thrombosis	• Inhibition of thrombus formation and fibrin generation and platelet aggregation	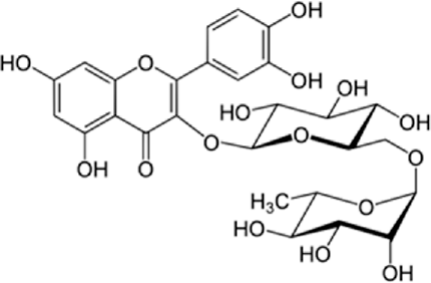
			0–100 μM			• Selective inhibition of PDI	
Zhang et al. (2013a)	*in vivo* and *in vitro*	Ghrelin	10^–8^ mol/kg/day	4 days	Apoptosis	• Inhibition of myocardial ER stress and	
			Every 12 h			• Protection of the heart against ER stress-induced apoptosis by activating AMP-activated protein kinase	
Tang et al. (2017)	*In vitro*	Naringenin	0–160 μM	24 h	Hypoxia/reoxygenation-induced apoptosis and cytotoxicity	• Amelioration of hypoxia/reoxygenation-induced endoplasmic reticulum stress-mediated apoptosis in H9c2 myocardial cells	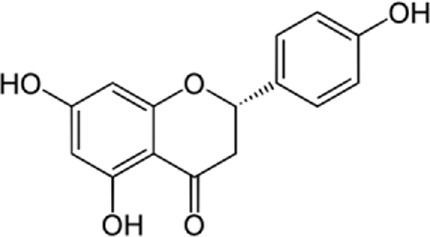
Qian et al. (2021)	*In vivo*	Icariin	10–40 mg/kg per day	2 weeks	Cardiomyocyte apoptosis	• Enhancement of left ventricular function and an increase in stroke output and ejection fraction	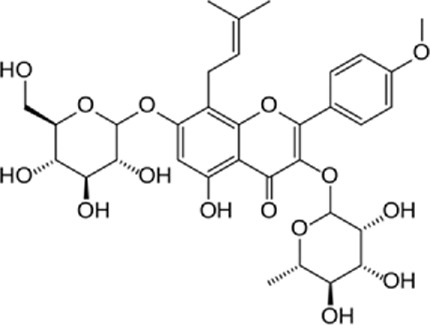
						• Prevention of ER stress-induced apoptosis	
Zhang et al. (2013b)	*In vitro*	Icariin	0–20 μM	30 min	Cardiac H9c2 cells apoptosis	• Protection of Rat Cardiac H9c2 Cells from Apoptosis	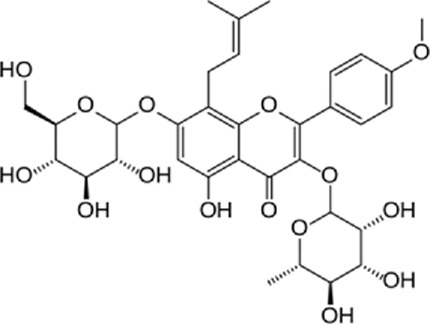
						• Inhibiting Endoplasmic Reticulum Stress	
Shen et al. (2014)	*In vitro*	Baicalin	0–50 µM	24 h	ER stress-induced apoptosis of cardiomyocytes	• Protection of cardiomyocytes from ER stress-induced apoptosis *via* CHOP/eNOS/NO pathway	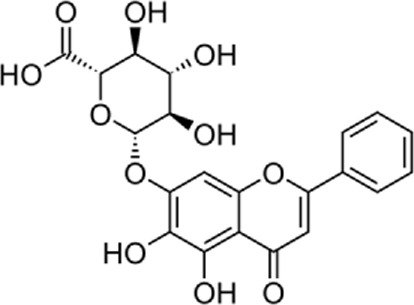
Yu et al. (2019a)	*In vitro* and *in vivo*	Naringenin	50 mg/kg/d	5 days	H9c2 cardiomyoblasts and MI/R-injured rat heart	• Increased myocardial cGMP	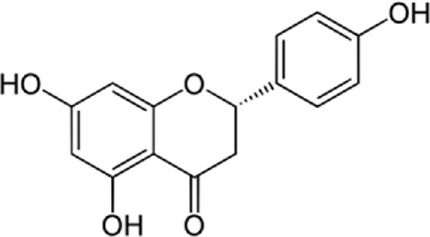
						• Upregulated PKGIα expression	
						• Higher antioxidant enzyme expression	
						• Decreased myocardial oxidative stress levels	
Kim et al. (2008)	*In vitro* and *in vivo*	Kaempferol	10 µM	20 min	MI/R-injured H9c2 cardiac muscle cells	• Increase in Bcl-2 (anti-apoptotic protein) expression	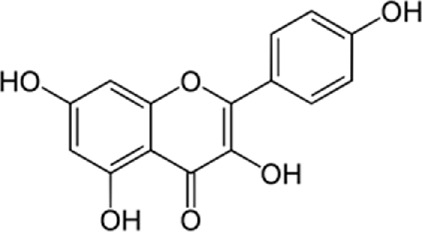
						• Decrease in Bax (apoptotic protein) expression	
						• Down-regulation of proteins involved in ER stress	
						• Improved the post-ischemic left ventricular end-diastolic pressure and also left ventricular diastolic pressure	
Zhang et al. (2019b)	*In vivo*	Nobiletin	15, 30, and 45 mg/kg	Pre-treatment	MI/R-injured rat heart	• Downregulated mRNA and protein levels of ER stress-related signal molecules including GRP78, caspase-12, and CHOP	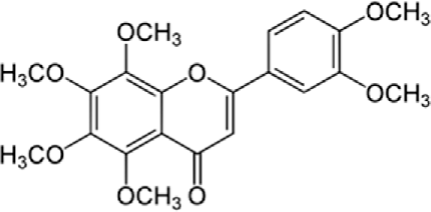
						• Increased levels of p-PI3K and p-AKT	
						• Mediated ER stress by PI3K/AKT pathway	
Feng et al. (2018)	*In vitro*	Apigenin	NR	Pre-treatment	MI/R-injured rat cardiomyocytes	• Enhanced cardiac performance	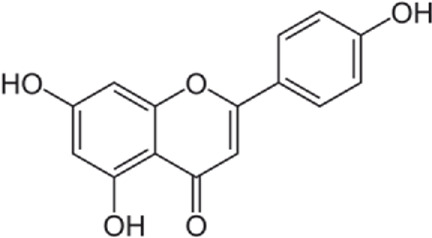
						• Reduced ER stress by stimulating the AMPK signaling pathway	
						• Reduced cell apoptosis	
						• Improved cell viability	
Shu et al. (2019)	*In vitro*	Dihydroquercetin	5, 10, 20 µM	20 min	MI/R-injured rat cardiomyocytes	• Inhibited the apoptotic pathways by decreasing CHOP and p-JNK	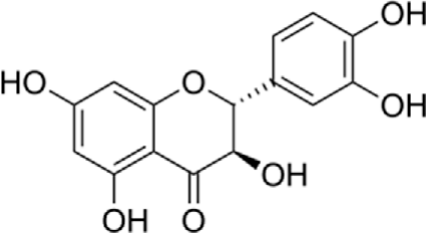
						• Postpone the onset of ER stress by decreasing (P-EIF2), PERK and GRP78	
						• Stimulated the expression of HO-1	
						• Increased Nrf2 binding to antioxidant response elements	
Kim et al. (2010)	*In vitro*	Flavonoids	10 µM biochanin A, 1 µM daidzein, 25 µM genistein, 10 µM luteolin, 50 µM quercetin or 50 µM rutin	30 min	MI/R-injured rat cardiomyocytes	• Raised the expression of Bcl-2	Many flavonoids
						• Reduced the Bax	
						• Decreased the glucose-regulated protein-78, inositol-needing protein-1, X-box binding protein 1, C/EBP-homologous protein, and phosphor-eukaryotic initiation factor 2α	
Zhu et al. (2017b)	*In vitro*	Luteolin	8 µM	12 h	MI/R-injured rat cardiomyocytes	• Increased SERCA2a activity	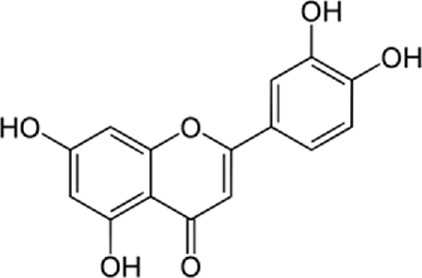
						• Decreased the inhibitive results of the p38 pathway	
Badawy Khair and Mohammed, (2021)	*In vivo*	Silymarin	NR	3 months	Atherosclerosis	• Reduced loss and disruption of Purkinje cell layer with pyknotic nuclei	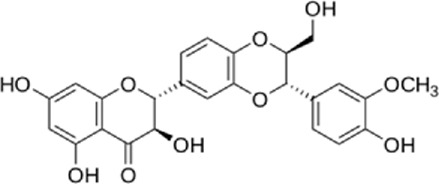
						• Reduced dilated cisternae of rough ER	
						• Prevented increase in GFAP, Cox-2 immunoreactivity	
Liew et al. (2003)	*In vitro*	Genistein	40 µM	5 min	Cardiomyocyte contraction	• Stimulated myocyte contraction	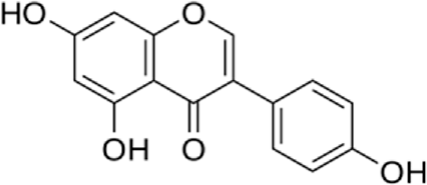
						• Inhibited the primary stimulus of cell contraction namely the L-type Ca2+ current	
						• Enhanced the SR Ca2+ load	
						• Transient increase in Ca2+ through Na+/Ca2+ exchanger dysfunction	
						• Influenced the phosphorylation of phospholamban	
						• Speeded up SR release by affecting the ryanodine receptor	
Feng et al. (2012)	*In vitro*	EGCG	1–100 µM	5 min	Cardiomyocyte contraction	• Increased the contractility of unchanged murine myocytes	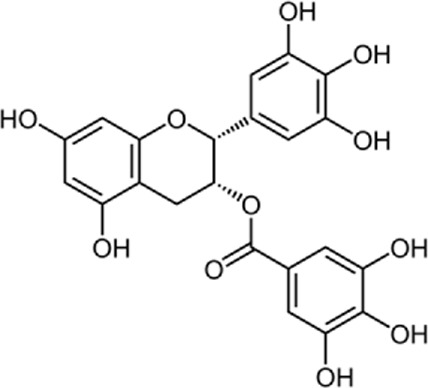
						• Raised SR Ca2+ content	
						• Electrically stimulated Ca2+ transients	
						• Inhibited the Na+/Ca2+ exchanger	
						• Ca2+-ATPase, Na + -K + ATPase, and Na + -H+ exchanger, were not affected	
Hamaguchi et al. (2021)	*In vivo*	Quercetin	10–30 µM	4–6 weeks	Diastolic dysfunction	• Accelerating myocardial relaxation through ER Ca2+-ATPase activation	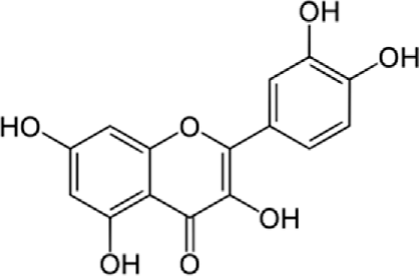
Zhou et al. (2016)	*In vivo* and *in vitro*	Flavonoids of Astragalus (TFA)	5–50 mg/kg	7 days	Viral myocarditis	• Preventing down regulation of ER chaperone calumenin expression and rescuing calumenin interaction and SERCA2	
Kim et al. (2008)	*In vitro*	Kaempferol	10 μM	19:50′	Ischemic/Reperfusion induced Cardiac Dmage	• Decreased apoptosis of heart muscle cells by increasing the anti-apoptotic protein BCL2 and also decreasing the expression of ER stress proteins	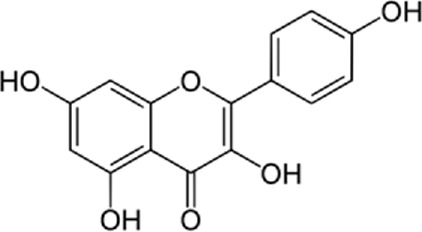
Qu et al. (2015)	*In vivo*	Wogonin	.1–100 µM	3:50′	Hypertension	• inhibition of both intracellular Ca2+ release and extracellular Ca2+ influx	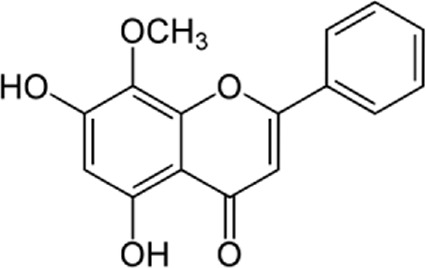
Xu et al. (2019)	*In vivo* and *in vitro*	Naringenin	100 mg/kg/d	12 weeks	Atherosclerosis	• Cholesterol efflux regulator through the ATF6 branch of ER stress and PI3K/AKT pathway	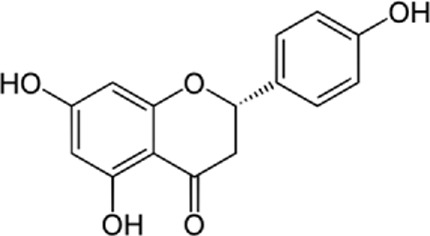
Dai et al. (2017)	*In vivo*	Baicalin	2 ml of 100 mg/kg daily	4 weeks	Myocardial fibrosis	• Reduction of ER stress and myocardial apoptosis and reverting left ventricular remodeling	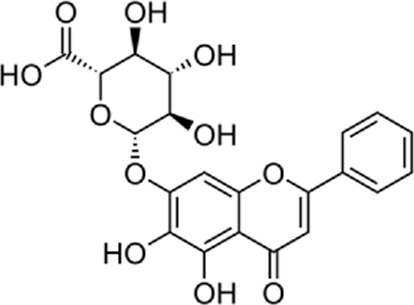
Ge et al. (2019)	*In vivo* and *in vitro*	Fisetin	40 mg/kg daily	16 weeks	Cardiac dysfunction	• Prevention of myocardial inflammation and fat deposition and cardiomyopathy by inhibiting ER signaling	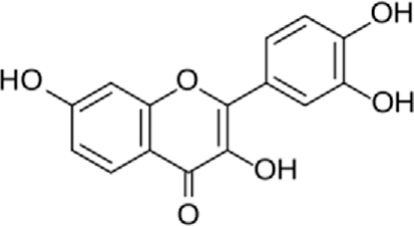
Wu et al. (2019)	*In vivo*	Icariside II	4–16 mg/kg daily	13 weeks	Hypertensive heart disease	• Prevention of ER-induced hypertensive disease by reducing cardiomyocyte apoptosis through inhibition of the PERK/ATF-4/CHOP signaling pathway	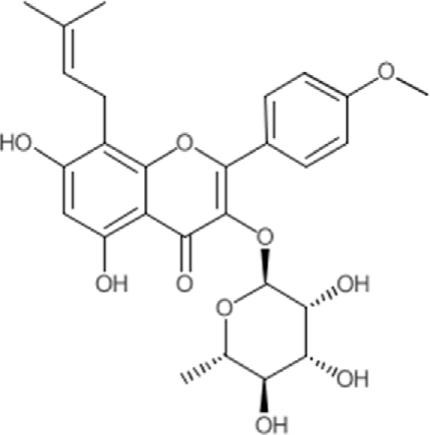
Ashokkumar et al. (2018)	*In vivo*	Vitexin	1.5 mg/kg.b.wt	30 days	Myocardial injury	• Enhanced cardioprotective effects by coordinated activation of ER stress	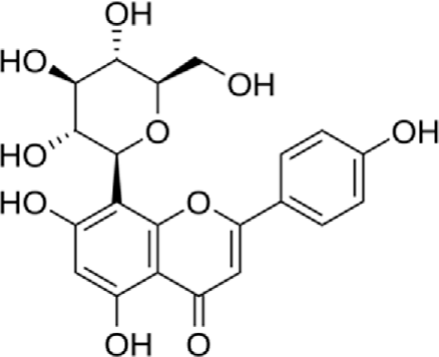

## 5 Cardiomyopathy

### 5.1 Quercetin

In an animal study, Cai et al. (2015) supported the role of quercetin in preserving rat hearts with post-myocarditis dilated cardiomyopathy. They had given the intervention group of rats quercetin at a dose of 10 mg/kg. Interestingly, they found reduced myocardial levels of fibrosis and ER stress in the intervention group compared to the vehicle-treated dilated cardiomyopathy rats (control group). Furthermore, the cardiac function and myocardial size were notably protected in the rats treated with quercetin in contrast to the control group. Surprisingly, the quercetin-treated rates showed considerable suppression of the myocardial endothelin-1 in addition to mitogen-activated protein kinases. In addition, Chang et al. (2021) conducted an *in vitro* study to evaluate the effects of quercetin on oxidative stress induced by hypoxia in human cardiomyocyte cell cultures. They found that quercetin could inhibit endoplasmic reticulum stress (ERS) by regulating silent information regulator protein 1 (SIRT1), which governs the ERS, apoptosis, oxidative stress, and also through regulating transmembrane BAX Inhibitor Motif-including protein 6 (TMBIM6), which has a protective function against cell death.

### 5.2 Nobiletin

Zhang et al. (2017b) conducted an *in vivo* and *in vitro* study on mice who underwent aortic banding (AB) operation to induce cardiac hypertrophy. The study group was remedied with 50 mg/kg of Nobiletin for 4 weeks. Based on the study results, nobiletin can prevent cardiac hypertrophy by reducing NADPH oxidase (NOX) 2 and NOX4 expression. Moreover, nobiletin could alleviate ER stress by inhibiting NOX2 in neonatal rat cardiomyocytes (NRCMs).

## 6 Autoimmune myocarditis

### 6.1 Quercetin

In a study by Arumugam et al. (2012), the effects of quercetin on the advancement of practical autoimmune myocarditis were evaluated. They found suppressed ER stress and oxidative stress through endothelin-1/MAPK signaling. Moreover, they showed that treatment with quercetin can suppress both the mitogen-active protein kinases (MAPK) and the myocardial endothelin-1. Consequently, therapy against the advancement of experimental autoimmune myocarditis (EAM) to dilated cardiomyopathy (DCM) affects the modulation of the MAPK signaling cascade. To investigate this hypothesis (Martinet and Kockx, 2001), an oral regime of quercetin (10 mg/kg/day) in rats with EAM induced by porcine heart myosin was conducted. It was found that the post-myocarditis rats travailed from increased ER stress and myocardial fibrosis. Quercetin showed considerable suppression of myocardial expressions of growth arrest, glucose-regulated protein 78 (GRP78) levels, and DNA damage-inducible factor 153 (GADD153). In addition, quercetin reduced the cytosolic level of cytochrome C which elevates apoptosis and therefore plays its protective role against adverse cardiac remodeling due to prolonged ER stress during DCM. Furthermore, quercetin significantly suppressed myocardial endothelin-1 (ET-1) and MAPK which causes EAM progression. All in all, quercetin plays its cardioprotective role against EMA through the modulating MAPK signaling cascade (Arumugam et al., 2012).

## 7 Thrombosis

### 7.1 Quercetin

**FIGURE 1:**
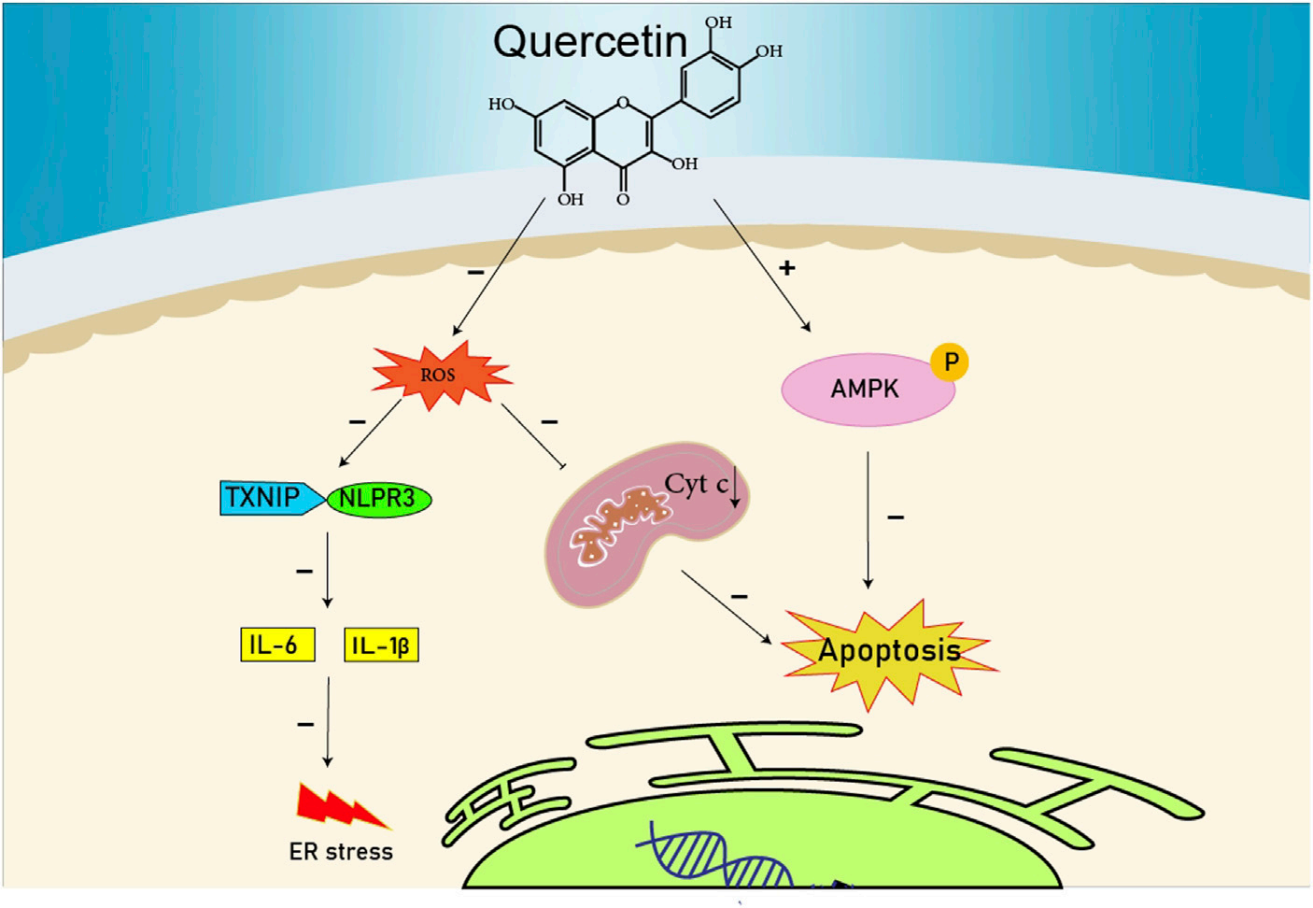
The reduction of ROS production by quercetin decreases TXNIP and NLPR3, which in turn results in a reduction of endoplasmic reticulum stress by reducing IL1B and IL6. On the other hand, the reduction of ROS reduces the production of Cyt c in the mitochondria, which ultimately reduces apoptosis. The compound also increases AMPK phosphorylation in the endothelial cells, causing an increase in NO production and eventually a decrease in apoptosis.

Quercetin-3-O-rutinoside, abundant in common foods, inhibits protein disulfide isomerase (PDI) and prevents thrombus formation *in vivo* and *in vitro*. Quercetin-3-O-rutinoside inhibits platelet aggregation *in vitro* and prevents injury-caused fibrin construction on endothelial cell monolayers (Figure 1) (Jasuja et al., 2012). In a laser-caused mouse model of thrombus formation, quercetin-3-O-rutinoside prevented fibrin formation and platelet accumulation at concentrations of .5 mg/kg (Jasuja et al., 2012). Quercetin-3-O-rutinoside inhibited thrombus formation in a ferric chloride-caused thrombus formation model. Intravenously quercetin-3-O-rutinoside and also oral compounds were antithrombotic *in vivo*. Notably, these combinations were antithrombotic at concentrations less than those used by individuals receiving over-the-counter formulations of quercetin-3-O-rutinoside. Quercetin-3-O-rutinoside inhibited purified PDI with an IC50 of ∼6 μM and bound PDI underflow with a Kd of ∼3 μM (Jasuja et al., 2012). They showed that just flavonoids with a 3-O-glycosidic link in the 3 positions of the C ring blocked PDI action. The fact that just glycosylated quercetins are active against PDI is critical when evaluating the possible toxicity of these combinations. This glycoside decreases the cell permeability of quercetin. Therefore, the exact chemical moiety that causes a quercetin analog active against PDI, inhibits its entrance into cells, which may be the cause of the low toxicity (Flaumenhaft, 2013; Chtourou et al., 2015; Bekendam and Flaumenhaft, 2016).

## 8 Myocardial cell death

### 8.1 Naringenin

Tang et al. (2017) showed that naringenin reduces H9c2 myocardial cell death due to ER apoptosis in an *in vitro* study. In this study, they found that naringenin reduces ER-caused apoptosis in H9c2 myocardial cells in several ways. Naringenin enhanced the expression of an anti-apoptotic protein called Bcl-2 and reduced the caspase-3 activity, Cytochrome c, and Bax expression as a pro-apoptotic protein (Kiraz et al., 2016; Qin et al., 2016). In addition, naringenin reduced the expression of ER stress-related proteins containing cleaved caspase-12, glucose-regulated protein 78, and C/EBP homologous protein (Miyazaki et al., 2011; Tao et al., 2011; Zhang et al., 2013a). Finally, activating transcription phospho-extracellular regulated protein kinases, inositol-requiring enzyme-1a, and factor 6, which are the three signaling pathways related to ER stress, were inhibited by naringenin (Ron and Walter, 2007; Wu et al., 2015; Yu et al., 2016). All these results showed that naringenin improves hypoxia/reoxygenation-induced apoptosis and cytotoxicity by moderating signaling pathways associated with ER stress.

### 8.2 Icariin

Qian et al. (2021) assessed the protection activity of Icariin against cardiomyocyte apoptosis due to hypertension-induced damage in an *in vivo* study. In this study, spontaneously hypertensive rats were selected and orally administered Icariin at the concentration of 10, 20, and 40 mg/kg per day in the study group and water in the control group for 2 weeks. Treatments with Icariin showed enhanced left ventricular function and increased stroke output and ejection fraction. The hypertrophy of left ventricular cardiomyocytes declined and the expression of ERS-induced proteins such as ATF-6, ATF-4, GRP78, PERK, CHOP, Caspase 12, DR5, c-JUN, and ASK-1 decreased. Consequently, Icariin prevented ERS-induced apoptosis (Qian et al., 2021). Moreover, Zhang et al. assessed *in vitro* effects of Icariin on the apoptosis induced by ERS. In this study, H9c2 rat cardiomyoblast cells were exposed to tunicamycin (ER stressor) and they were pretreated with Icariin (2.5, 5, 10, and 20 μm) for 30 min in advance. They reported that Icariin reduced the formation of reactive oxygen species (ROS) and caspase-3 activity. Moreover, ER markers’ levels and mitochondrial membrane potential decreased. In conclusion, this study showed the protective efficacy of Icariin in the apoptosis (Zhang et al., 2013b).

### 8.3 Baicalin

Shen et al. (2014) determined the effects of Baicalin on cardiomyocyte apoptosis induced by ER stress in an *in vitro* study. The result of this study indicated that Baicalin possesses anti-oxidant and anti-apoptotic properties and it can reduce ER stress-induced cardiomyocyte apoptosis through the CHOP/Enos/NO pathway, due to decreasing the expression of CHOP, caspase-3, and Enos, JNK.

## 9 Myocardial ischemia-reperfusion injury

### 9.1 Naringenin

Yu et al. (2019a) investigated the effects of naringenin on myocardial ischemia-reperfusion damage and cyclic guanosine monophosphate-PKGI alpha. They found that the naringenin-treated group showed antioxidant enzyme expressions and decreased myocardial oxidative stress levels during myocardial ischemia-reperfusion injury. Previous studies showed that naringenin had antioxidative impacts and developed cardiac functional recovery after the injury. These data also demonstrated that naringenin, by its antioxidant impacts, may protect against ischemic heart disease. Previously, it was discovered that cGMP-PKG signaling affected myocardial ER function and decreased ER stress levels in stress situations (Han et al., 2012; Ramprasath et al., 2014; Subburaman et al., 2014; Chtourou et al., 2015; Da Pozzo et al., 2017). In conclusion, both *in vivo* and *in vitro* studies showed that naringenin increased myocardial cGMP content and upregulated PKGIα expression.

### 9.2 Kaempferol

**FIGURE 2:**
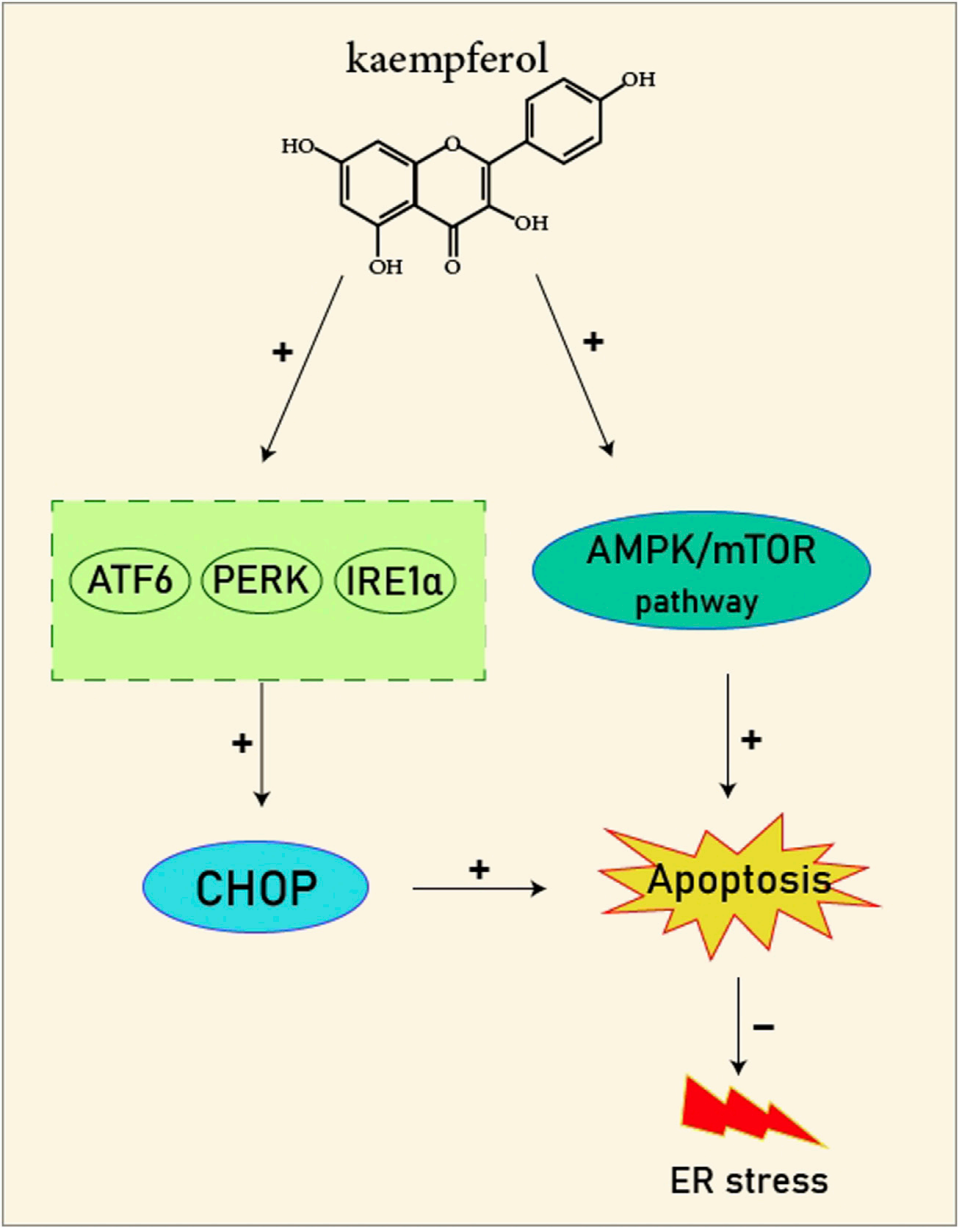
Kaempferol induces CHOP through IRE1a, PERK, ATF6 and also induces AMPK/mTOR, which induce apoptosis and as result reduce ER stress.

In a study by Kim et al. (2008), *in vitro* effect of Kaempferol treatment on H9c2 cardiac muscle cells was assessed. The study group was perfused with a 10 µM concentration of Kaempferol during the pre-ischemia period for 20 min followed by the post-ischemia period for 50 min. They found that Kaempferol, as a treatment, could lead to a considerable increase in Bcl-2 (anti-apoptotic protein) expression and a decrease in Bax (apoptotic protein) expression. In addition, the downregulation of proteins involved in ER stress was observed in both *in vivo* and *ex vivo* experiments. The comparison between the study and control group (vehicle) in the *ex vivo*-Langendorff experiment showed that kaempferol could improve the post-ischemic left ventricular end-diastolic pressure and also left ventricular diastolic pressure noticeably, followed by 20, 30, 40, and 50 min of reperfusion (Figure 2).

### 9.3 Nobiletin

Zhang et al. (2019b) assessed the effects of Nobiletin on reperfusion and myocardial injury. In this study, rats were treated with Nobiletin (15, 30, and 45 mg/kg) followed by the induction of myocardial ischemia and reperfusion injury. The study represented that mRNA and protein levels of ER stress-related signal molecules including GRP78, caspase-12, and CHOP are downregulated by Nobiletin. In contrast, the levels of p-PI3K and p-AKT are increased. Finally, the attenuation of myocardial apoptosis caused by PI3K/AKT-mediated ER stress was observed.

### 9.4 Apigenin

Feng et al. (2018) studied the Apigenin-7-O-β-D-(-6''-p-coumaroyl)-glucopyranoside (APG) cardioprotective mechanism and the role of ER stress in myocardial ischemia/reperfusion in rat hearts. They concluded that pretreatment with Apigenin enhances cardiac performance and reduces ER stress by stimulating the AMPK signaling pathway. This signaling pathway has several beneficial activities such as reducing cell apoptosis, enhancing cardiac performance, and improving cell viability.

### 9.5 Dihydroquercetin

Shu et al. (2019) evaluated the impact of Dihydroquercetin (DHQ) on ER stress-induced apoptosis in rat cardiomyocyte cell cultures. Their study showed that DHQ inhibits the apoptotic pathways by decreasing the expression levels of pro-apoptotic proteins such as C/EBP homologous protein (CHOP), Caspase-12, and c-junk-terminal kinase (p-JNK). DHQ can also postpone the onset of ER stress by decreasing the expression levels of Phosphorylated eukaryotic translation initiation factor 2 (P-EIF2), protein kinase-like Endoplasmic reticulum kinase (PERK), and Glucose-regulated protein 78 (GRP78), and stimulating the expression of Heme Oxygenase-1 (HO-1) which is a cytoprotective enzyme, and increasing nuclear factor-erythroid factor 2-related factor 2 (Nrf2) binding to antioxidant response elements which play a main role in the expression of HO-1. In total, it can be concluded that DHQ can significantly decrease ER stress and its consequent apoptosis.

### 9.6 Flavonoids

Kim et al. (2010) investigated the protective effect of flavonoids on reperfusion/ischemia-cause cardiac impairment in an *in vitro* study. They found that Flavonoids, as a treatment, notably raised the expression of an anti-apoptotic protein (Bcl-2) but reduced the proapoptotic protein called Bax. The flavonoids decreased the expression levels of ER stress proteins, glucose-regulated protein-78, triggering transcription factor 6α, inositol-needing protein-1, X-box binding protein 1, C/EBP-homologous protein, and phosphor-eukaryotic initiation factor 2α. This study indicated that cardiac reperfusion/ischemia was associated with the ER stress reaction, in which mitochondrial Bcl-2 proteins were affected too.

### 9.7 Luteolin

Zhu et al. (2017b) used a p38 mitogen-activated protein kinase inhibitor (SB203580) and luteolin to determine whether SERCA2a is involved and impacts the negative cardiac role caused by p38 MAPK during myocardial I/R injury. This study showed that luteolin increases SERCA2a activity to enhance systolic/diastolic role during Ischemia/Reperfusion in rat hearts and cardiomyocytes by decreasing the inhibitive results of the p38 pathway (Zhu et al., 2017b).

## 10 Atherosclerosis

### 10.1 Silymarin

A study by Badawy Khair and Mohammed (2021) compared the neuroprotective effects of silymarin and Co Q10 on sixty adult male albino rats suffering from atherosclerosis. They divided rats into four classes: Group one (control group), Group two (atherosclerotic group), Group three (atherosclerotic Co Q10 treated group), and Group four (atherosclerotic Silymarin treated group). Three months later they processed cerebellar cortices that demonstrated widened cisternae of rough ER and disruption and loss of the Purkinje layer with pyknotic nuclei. Thus, Silymarin supplementation had a neurological protecting function in atherosclerosis. Silymarin is perhaps the perfect neuroprotective mechanism and an unexplored method for decreasing many neurological disorders in high-risk people (Badawy Khair and Mohammed, 2021).

## 11 Cardiomyocyte contraction

### 11.1 Genistein

Liew et al. (2003) conducted a study on a guinea pig and showed that genistein increases cardiac excitation-contraction coupling. The most important result of this study is the stimulation of myocyte contraction in the guinea pig by genistein while inhibiting the primary stimulus of cell contraction namely the L-type Ca^2+^ current (Liew et al., 2003). Their examinations with caffeine-induced contractions showed that genistein enhances the sarcoplasmic reticulum (SR) Ca^2+^ load. The following mechanisms are involved: Genistein causes a transient increase in Ca^2+^ through Na^+^/Ca^2+^ exchanger dysfunction, resulting in increased calcium reabsorption by the SR (Bers and Bridge, 1989; Bers and Berlin, 1995). Furthermore, it is conceivable that genistein influences the phosphorylation of phospholamban, raising the Ca^2+^ affinity of the SR Ca2+ pump. Moreover, genistein can speed up SR release by affecting the ryanodine receptor. Altogether, their findings support the effect of genistein on the cardiovascular system as a positive cardiac inotrope.

### 11.2 Catechin

In a study conducted by Feng et al. (2012) nanomolar concentrations of epigallocatechin 3 gallate (EGCG) notably increased the contractility of unchanged murine myocytes by raising SR Ca^2+^ content, electrically stimulated Ca^2+^ transients, and ryanodine receptor type 2 channel opening possibility. Voltage clamp experimentations showed that epigallocatechin-3-gallate notably inhibits the Na^+^/Ca^2+^ exchanger. However, other Na^+^ and Ca^2+^ handling proteins like Ca^2+^-ATPase, Na^+^-K^+^ ATPase, and Na^+^-H^+^ exchanger, were not affected by EGCG. Therefore, nanomolar EGCG rises contractility in unchanged myocytes. These findings should be further evaluated and may supply a new therapeutic approach to enhancing the heart’s contractility.

## 12 Cardiomyocyte relaxation

Hamaguchi et al. (2021) examined the lusitropic effects of quercetin on isolated ventricular myocardia from normal and Streptozotocin-caused diabetic mice. In this study, they showed that quercetin can speed up myocardium relaxation and detected the carrier involved by selective Sarco–endoplasmic reticulum Ca^2+^ pump (SERCA) and Na^+^–Ca^2+^ exchanger (NCX) blockers. The SERCA is the main carrier to absorb Ca2+ from the cytoplasm to the SR (Choi et al., 2002; Belke and Dillmann, 2004), while cyclopiazonic acid (CPA) is a SERCA inhibitor (Takahashi et al., 1995). These results indicated that quercetin has positive lusitropic results on the myocardium. The action was blocked by CPA, which indicates that it is done by activating SERCA. Quercetin decreased the contractile strength and tended to speed up the contraction rate. Quercetin was reported to block the ryanodine receptor (Carrasco-Pozo et al., 2012) and the L-type Ca^2+^ channel (Castillo et al., 2018; Liang et al., 2020). Its blockage by quercetin may reduce the Ca^2+^ concentration and the moment to achieve it. Altogether, the quercetin effect on the myocardium is diverse from agonists of β-adrenoceptor, which present both positive lusitropic and inotropic effects (Namekata et al., 2018). This suggests that quercetin is less likely to enhance the myocardial need for O_2_, which may be a benefit in the cure of failing myocardium. Quercetin caused a little reduction in the contractile strength and the contraction duration and speeded up the relaxation, which depends on concentration. The quercetin-caused speed of relaxation was completely blocked by CPA.

## 13 Viral myocarditis

### 13.1 Flavonoids

The study by Zhou et al. (2016) attempted to show the relationship between the pathophysiology of viral myocarditis and total flavonoids of Astragalus and identify the possible cardioprotective effects of total flavonoids of Astragalus (TFA). CoxsackievirusB3 (CVB3) infection caused the cardiac malfunction and histopathological estimation demonstrated myocyte degeneration. Intraperitoneal injections of total flavonoids of Astragalus with CVB3 rescued functional and also morphological parameters. *In vitro* studies in HL-1 cells showed acute treatment with high doses of TFA develops SERCA2 and calumenin association, the association that was reduced during CVB3 infected cells. The protective effect of TFA was evaluated by treating animals simultaneously with TFA at a dose of 20 mg/kg and CVB3. Medium doses of TFA prevented the loss of calumenin, as mRNA levels were higher in animals that were treated by TFA and CVB3 compared to animals infected with CVB3 alone. This effect was protein specific as neither CVB3 infection nor TFA treatment changed the protein levels of the ER Ca^2+^-ATPase (SERCA2), showing that the cardio-protective effect of TFA in a mouse model of viral myocarditis is through mediating the translation and transcription of proteins that are applied in ER stress and the UPR pathway rather than regulating of calcium mobilizing proteins. Interestingly, high dosages of TFA treatment did not rescue the deficit in calumenin mRNA levels, which is emphasizing the limited sufficient dose range or therapeutic window of this flavonoid. In conclusion, the study stressed the therapeutic potential of TFA in treating viral myocarditis, suggesting that this compound conserves pathogen-caused cardiomyopathy by regulating those elements involved in ER stress and calcium homeostasis ER stress in cardiomyocytes. For the first time, TFA was shown to prevent loss of mRNA and protein levels of calumenin and also maintained the connection of this protein with the Sarco-endoplasmic reticulum Ca^2+^-ATPase (SERCA2) (Zhou et al., 2016). Xuan et al. (2016) evaluated the impact of whole flavonoids on the expression of ER connexin 43, calumenin, and chaperone in suckling mouse myocardium heart muscle inflammation induced by CVB3. They randomly divided the initial culture of mouse myocardium cells into total flavonoids, CVB3 infected group, and control group. CVB3 infection perhaps induces ER stress of myocardium cells by decreasing the expression of CX43 and calumenin and raising the expression of GRP78. The results of this investigation may be closely linked to the results of anti-arrhythmia with virus-related myocarditis yielded by CVB3 (Xuan et al., 2016).

## 14 Cardioprotection

### 14.1 Kaempferol

Kaempferol (3,4/,5,1-tetrahydroxyflavoune) is a yellow flavonoid compound that is seen in different fruits and vegetables like broccoli, cabbage, tea, etc., (Georgiev et al., 2014). Kaempferol is often administrated in traditional medicine because of its multiple therapeutic and biological activities including antioxidants (Du et al., 2018; Wu et al., 2018), anti-obesity (Torres-Villarreal et al., 2019), and cardiovascular protection (Zhou et al., 2015b; Suchal et al., 2016; Xiao et al., 2016; Suchal et al., 2017). Targeting ER stress pathways seems to be useful in related diseases. Meanwhile, among the traditional herb medicine, kaempferol as a flavonoid appears to be hopeful to adjust ER stress and autophagy and display protective impacts on cells. Some reports are showing the ability of kaempferol in influencing ER stress and autophagy. Briefly, kaempferol adjusts autophagy in non-cancerous cells to protect cells against dysfunction.

Kaempferol can exert a modulatory effect on ER stress. Upon ER stress, protein kinase RNA-like endoplasmic reticulum kinase (PERK), inositol-requiring enzyme 1 (IRE1α), and activating transcription factor 6 (ATF6) undergo activation to decrease a load of stress, and kaempferol also prompt these pathways. In addition, kaempferol produces CHOP through IRE1α, PERK, and ATF6 to trigger apoptotic cell death and reduce ER stress. Another key pathway for ER stress blockage by kaempferol is the prompting of 78-kDa glucose-regulated protein (GRP78) (Ashrafizadeh et al., 2020). Markedly, during stress, autophagy is activated to reduce the load of stress and loss of autophagy in performing this target causes the induction of apoptosis. Following this strategy, kaempferol positively impacts the AMPK/mTOR (mammalian target of rapamycin) signaling pathway to yield autophagy, leading to ER stress inhibition. Although, if the induced autophagy could not reduce the stress, kaempferol activates CHOP *via* pathways leading to apoptosis (Ashrafizadeh et al., 2020). In a study by Kim et al. (2008), cardioprotective effects of a 10 μm dosage of Kaempferol on the H9c2 cardiac muscle cells were observed. Major outcomes were protection against ischemic/reperfusion damage by down-regulation of ER stress-related markers like ATF6, GRP78, ATF6, x-box binding protein 2 (XBP-2), CHOP, and IRE1. To the best of our knowledge, ATF6, IRE1α, and PERK are considered the three main upstream regulators of ER stress (Ghobadi et al., 2017; Najafi et al., 2017; Rezapoor et al., 2017). It was recommended that kaempferol is a naturally occurring compound that has inhibitory and stimulatory function on ER stress (Ashrafizadeh et al., 2020).

### 14.2 Wogonin

Qu et al. (2015) reported that wogonin prevented the increase of Ca^2+^ initiated by KCl (60 mm) after exhausting calcium stored in sarcoplasmic and endoplasmic reticula with thapsigargin (1 μm) or by ATP (100 μm) in VSMCs. Wogonin is a flavone and has a mixture of cardiovascular defensive effects. This report showed that wogonin has therapeutic potential for cerebrovascular and cardiovascular diseases. Although, it was informed that little wogonin was uncovered in rat plasma after an intragastric regime of wogonin (5 mg/kg).

### 14.3 Naringenin and naringin

Different *in vitro* and *in vivo* studies have verified the positive effects of naringenin on the prevention of atherosclerosis progression. In an experiment, favorable effects of naringenin and naringin as powerful anti-atherogenic compounds were detected in rabbits fed a high-cholesterol regime. It has been discovered that the anti-atherogenic result is mainly correlated to a reduced hepatic acyl-coenzyme A, cholesterol acyltransferase activity, downregulation of monocyte chemotactic protein-1 (MCP-1), and VCAM-1 genes (Lee et al., 2001). The impacts of the naringenin cure against cardiac hypertrophy may be related to reducing angiotensin-converting enzyme 1 (ACE1) and angiotensin II expression in cardiac tissues (INVALID CITATION). Naringenin provided conservation against ischemia/reperfusion damage by stimulating mitochondrial biogenesis and protecting mitochondrial function by the AMP-activated protein kinase-sirtuin 3 signaling pathway (Yu et al., 2019b). Contrary to lipid accumulation, naringin protects the myocardium (Rajadurai and Mainzen Prince, 2006). In a confirmatory study, naringenin stimulated myocardial cGMP peroxisome proliferator-activated receptor gamma coactivator 1-alpha signaling *in vivo* and *in vitro* which was conducted to reduce oxidative stress and ER stress levels, in addition to inhibited myocardial apoptosis during Ischemia/reperfusion state (Yu et al., 2019a). Naringenin defended the heart *via* the activation of mitochondrial large-conductance calcium-activated potassium channel (mitoBK) and could establish cardioprotection in senescent H9c2 cardiomyoblasts (Testai et al., 2017). Naringenin has a potent defensive impact on the myocardial cells against age-related harm by modulating the ROS levels, the estrogen-associated pathway, and mitochondrial potassium channels (Da Pozzo et al., 2017). Xu et al. (2019) discovered the impacts and effects of naringenin on cardiovascular diseases and lipoprotein profiles *in vitro* and *in vivo*. The results indicated that naringenin decreased ER stress in atherosclerotic stations and the ER stress-ATF 6 paths were involved with the naringenin-inhibited lipid accumulation and foam cell-building.

## 15 Others

### 15.1 Modulating ER stress in cardiovascular disease

#### 15.1.1 Quercetin

Quercetin has been shown to reduce the creation of ROS impeding pyrin domain containing-3 (NLRP3), the NOD-like receptor family, and thioredoxin-interacting protein (TXNIP) inflammasome activation caused by palmitate. TXNIP and NLRP3 stimulate the induction of IL-6 and IL-1 β, which can cause ER stress. In addition, quercetin can promote basal AMPK activity by improving the phosphorylation of AMPK increasing nitric oxide (NO) production in endothelial cells, and preventing cell apoptosis through changing δψm (Wu et al., 2014). The inhibitory effects of 30, 40-dihydroxyflavonol (DHF) or 5,7-dideoxyquercetin on ER stress caused by tunicamycin were evaluated in the C57BLK/6 J mice’s aorta. DHF decreased the expression of GRP78 and CHOP, caspase-3 cleaved structure, and eif2α phosphorylation. Moreover, DHF decreased ROS generation and increased NO in aortic rings. An *in vitro* study showed that DHF reduced eIF2 *α* phosphorylation and GRP78 expression induced a rise in XBP1 splicing and induced apoptosis (Lau et al., 2018). Oral administration of quercetin (10 mg/kg/day) was found to prevent the exacerbation of experimental autoimmune myocarditis to dilated cardiomyopathy. Quercetin reduced the myocardial expression of GRP78 and GADD153. In addition, it suppressed the endothelin-1 (ET-1) and myocardial MAPK yielding the progression of experimental autoimmune myocarditis. Quercetin reduced the expression of mouse polyclonal antiosteopontin and TGF-ß 1 that emerge in fibrosis. It even decreased the cytosolic cytochrome C amount which was increased in the apoptosis (Arumugam et al., 2012). Therefore, based on these investigations, quercetin can be effective to decrease the impact of ER stress signaling ways and preserving against cardiovascular disease (Eisvand et al., 2022).

### 15.2 Blood pressure and left ventricular remodeling

#### 15.2.1 Baicalin

An *in vivo* study conducted by Dai et al. (2017) assessed how baicalin affects left ventricular remodeling and also changes blood pressure in renal hypertension rats. The control group administered .9% NaCl solution, while the study group received baicalin at the concentration of 100 mg/kg with .9% NaCl solution once a day for 4 weeks. The result of the study showed significant positive effects on left ventricular remodeling and a noticeable decline in the expression of fibrosis-related factors and caspase-3. Moreover, the expression of glucose-regulated proteins including GRP78 and GRP94, and also pro-apoptotic factors including CHOP and caspase-3 decreased in the study group receiving baicalin.

### 15.3 Insulin resistance and hyperlipidemia

#### 15.3.1 Apigenin

Wu et al. (2021) evaluated the role of apigenin in insulin resistance in mice. They found that apigenin could lower the levels of stearyl-CoA desaturase 1, sterol regulatory element-binding protein 2 (SREBP-2), sterol regulatory element-binding protein 1c (SREBP-1c), 3-hydroxy-3-methyl-glutaric CoA reductase, fatty acid synthase, and accumulation of lipid in the cells of the specimens, and thus leading to body weight loss. In addition, apigenin can reduce insulin resistance by decreasing ER stress. They concluded that apigenin can reduce hyperlipidemia, insulin resistance, body fat, and consequently diabetes and cardiovascular diseases.

### 15.4 Cardiac dysfunction

#### 15.4.1 Fisetin

A study by Ge et al. (2019) showed how fisetin and metformin remarkably suppressed metabolic stress-induced cardiomyopathy. Fisetin is amongst the most significant edible flavonoids with conceivably useful properties for human health (Khan et al., 2013; Shi et al., 2018; Yousefzadeh et al., 2018). Mice were selected at random to be fed an HFD (high-fat diet) and were given 4-PBA (4-phenyl butyric acid), fisetin, and metformin solution by gavage daily; this continued for 16 weeks. The therapeutic effects of fisetin and metformin on metabolic stress-induced heart injury were confirmed in HFD-challenged mice *in vivo*. Fisetin or metformin treatment noticeably reduced HFD-induced heart weight and heart weight to tibia length ratio. Furthermore, fisetin or metformin decreased IL-1β, IL-4, F4/80, IL-6, TNF-α, and CCL2 mRNA levels in cardiac samples from HFD-fed mice. Fisetin or metformin significantly recovered superoxide dismutase activity and lowered malondialdehyde levels in HFD-induced heart samples. The *in vitro* study confirmed that fisetin or metformin exposure exhibited an important role in easing PAL-caused oxidative stress, ER stress, inflammation, and dyslipidemia in cardiomyocytes and macrophages. At last, western blot analysis demonstrated that fisetin or metformin treatment supposedly prevents the expression of p-PERKT980, p-eif2α S51, pire1s724, XBP1, and CHOP in heart tissues from HFD-challenged mice. In conclusion, the results suggested that fisetin or metformin could reduce metabolic stress-caused cardiac inflammation, abnormal function, oxidative stress, and ER stress (Ge et al., 2019).

### 15.5 Hypertensive heart disease

#### 15.5.1 Icariside II

A study by Wu et al. (2019) aimed to discover whether Icariside II can inhibit ER stress-caused cardiomyocyte apoptosis through the PERK/ATF-4/CHOP signaling pathway. Hypertensive rats were separated into model groups and Icariside II groups at random. The rats in the Icariside II groups were intragastrically managed with Icariside II 4, 8, and 16 mg/kg from 14 to 26 weeks-age. At the end of the 26th week, cardiomyocyte apoptosis was analyzed and the levels of GRP78, PERK, ATF-4, and CHOP gene and protein were detected. *In vivo* study highlighted that the activation capacity of protein kinase RNA-like ER kinase was increased, and the expression levels of ATF-4 and CHOP protein were upregulated in spontaneously hypertensive rats, demonstrating that cardiomyocyte apoptosis caused by ER stress was through activating PERK. Icariside II weakened the phosphorylation capacity of protein kinase RNA-like ER kinase and reduced the expression of ATF-4 and CHOP, signifying its inhibitory effect on the PERK signaling pathway. To conclude, Icariside II prevents hypertensive heart disease by alleviating ER stress-induced cardiomyocyte apoptosis, and its mechanism is associated with the inhibition of the PERK/ATF-4/CHOP signaling pathway.

### 15.6 Myocardial injury

#### 15.6.1 Vitexin

The study by Ashokkumar et al. (2018) focused on the improvement of ER function *via* suppression of the extra ROS production with the raw flavonoid Vitexin. This study was designed to determine the impact of isoproterenol agonist b-AR moderated stimulus of ER stress and its joint activation of hippo signaling in the heart in the time of post-myocardial infarction. In brief, it showed the therapeutic ability of Vitexin against post-myocardial infarction and may pavSe the way for expanding a natural drug against post-myocardial infarction individuals as a non-invasive treatment (Ashokkumar et al., 2018).

## 16 Conclusion

Amongst all the groups of Flavonoids that have been reviewed in this study, Flavonols, Flavanones, and Flavones have been the most preferred groups; which include familiar cardioprotective compounds such as Quercetin, Kaempferol, Naringenin, Nobiletin, and Apigenin. Quercetin which might be the most known member of the Flavonol group and even Flavonoids in general could be found in red onion, kale, and many other vegetables. Studies showed that quercetin can decrease ER stress, either protecting against oxidative stress injuries or mitigating ER stress directly and the related apoptosis. Quercetin was protective in myocarditis by preventing mitochondria dysfunction and caspase activity inhibition in H9c2 cardiomyoblasts. Moreover, it could have been able to downregulate important ER stress markers such as GADD153, GRP78, and cytosolic Cytochrome C levels. Kaempferol is another subtype of flavonol and natural anti-oxidant that has been proven to have an inhibitory effect on ER stress proteins, such as GRP78, ATF-6alpha, and CHOP and an anti-apoptotic effect by regulating B-cl2 and Bax proteins, which can explain its impressive role in protection against ischemic heart disease. Naringenin, a Flavanone derived from grapefruit and herbs, also has been reported frequently to have anti-oxidant effects. It reduces ER stress levels by activating the cGMP-PKGIα signaling pathway. Since ER stress-associated apoptosis can be a consequence of myocardial ischemia/reperfusion injury, further in vivo-in vitro studies may demonstrate the pharmacological effects of this compound in myocardial ischemia/reperfusion patients. Nobiletin and Apigenin are both members of the Flavone group. *In vivo* experiments about cardiac hypertrophy have displayed inhibiting effects for Nobiletin on NOX4 and NOX2 expression, which can suppress ER stress. It can also attenuate the apoptosis that is mediated by ER stress by regulating PI3K/AKT pathway in myocardial ischemia/reperfusion injury. Apigenin is also beneficial in myocardial ischemia/reperfusion injuries and in modifying ER stress *via* the AMPK pathway. Some outstanding questions in the context of flavonoid intake in cardiovascular diseases remain for further studies. For instance, is there any place for flavonoids to show synergistic effects in combination with other cardiovascular medications? Does the consumption of flavonoids have significant side effects? Do any nano-formulated flavonoids exist? If yes, what are their advantages rather to conventional formulations? More pre-clinical investigations and further RCTs could be helpful to confirm Flavonoids’ therapeutic effect in clinical settings.
